# A Review of Mesoporous Silica Nanoparticle Delivery Systems in Chemo-Based Combination Cancer Therapies

**DOI:** 10.3389/fchem.2020.598722

**Published:** 2020-11-24

**Authors:** Ying Gao, Dongruo Gao, Jie Shen, Qiwen Wang

**Affiliations:** ^1^Department of Pharmacy, School of Medicine, Zhejiang University City College, Hangzhou, China; ^2^Department of Pharmaceutics, College of Pharmaceutical Sciences, Zhejiang University, Hangzhou, China; ^3^College of Chemical and Biological Engineering, Zhejiang University, Hangzhou, China; ^4^Department of Cardiology, The First Affiliated Hospital, Zhejiang University School of Medicine, Hangzhou, China

**Keywords:** mesoporous silica nanoparticles (MSNs), drug delivery systems (DDS), chemotherapy, combined cancer therapies, phototherapy, gene therapy

## Abstract

Chemotherapy is an important anti-tumor treatment in clinic to date, however, the effectiveness of traditional chemotherapy is limited by its poor selectivity, high systemic toxicity, and multidrug resistance. In recent years, mesoporous silica nanoparticles (MSNs) have become exciting drug delivery systems (DDS) due to their unique advantages, such as easy large-scale production, adjustable uniform pore size, large surface area and pore volumes. While mesoporous silica-based DDS can improve chemotherapy to a certain extent, when used in combination with other cancer therapies MSN based chemotherapy exhibits a synergistic effect, greatly improving therapeutic outcomes. In this review, we discuss the applications of MSN DDS for a diverse range of chemotherapeutic combination anti-tumor therapies, including phototherapy, gene therapy, immunotherapy and other less common modalities. Furthermore, we focus on the characteristics of each nanomaterial and the synergistic advantages of the combination therapies. Lastly, we examine the challenges and future prospects of MSN based chemotherapeutic combination therapies.

## Introduction

Despite the rapid development of medicine, the incidence and mortality of cancers are consistently rising and cancer remains one of the most terrible threats to human lives (Siegel et al., 2020). Traditional chemotherapy is one of the most common cancer treatments and is the most effective systemic treatment, playing an irreplaceable role in current treatment modality (Dai et al., 2016). However, the clinical application of chemotherapy is limited by several deficiencies: First, most chemotherapy drugs have poor aqueous solubility or short half-life *in vivo*, leading to low drug utilization. Second, chemotherapeutic drugs show poor tumor selectivity (Akhtar et al., 2014), resulting in the undifferentiated killing of both tumor and healthy cells. This non-targeted lethality not only reduces the therapeutic effect against carcinomas but also causes severe side effects. Last, is the multidrug resistance (MDR) induced by chemotherapy. MDR refers to the resistance of cancer cells to a variety of drugs which are structurally and functionally unassociated (Kong et al., 2017). This phenomenon is one of the primary causes of chemotherapy failure, leading to the recurrence of tumors, patient relapse, or even death (Wang J. et al., 2017).

**Figure 1:**
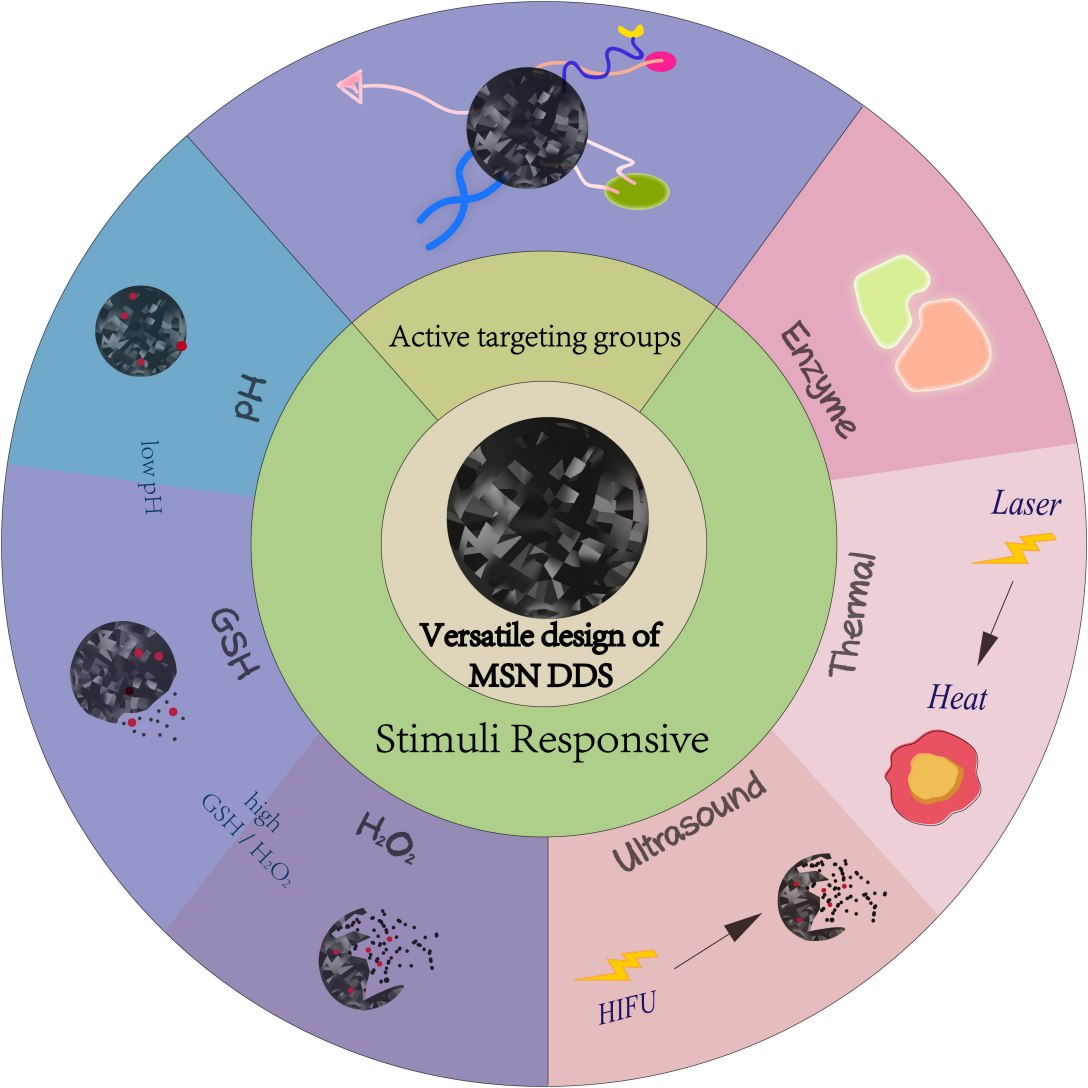
Versatile design of MSN DDS.

Recent years have witnessed many efforts to overcome the shortcomings of conventional chemotherapy. One of the most promising is the development of nano drug delivery systems (nano-DDS), which can increase the solubility and bioavailability of drugs, prolong the circulation time of drugs, increase the accumulation of drugs in tumor tissues, and improve the pharmacokinetic behavior *in vivo*, improving the curative effect of therapies while reducing the side effects (Mu et al., 2020). Common nanocarriers include polymers (Alsehli, 2020), liposomes (Allen and Cullis, 2013), dendrimers (Dias et al., 2020), inorganic nanoparticles like gold nanoparticles (GNPs) (Ajnai et al., 2014), and carbon nanomaterials (Chen D. et al., 2015). Among these nano-DDS, mesoporous silica nanoparticles (MSNs) are a class of materials that have garnered particular focused by many researchers, due to their facile large-scale production, adjustable uniform pore size (Bouchoucha et al., 2016), and large surface area and pore volume (Farjadian et al., 2019). These properties endow MSNs with good drug encapsulation efficiency and delivery, with uncomplicated preparation like the sol–gel “chimiedouce” methods in aqueous solutions (Croissant et al., 2018). Since silica-based materials have been considered safe by Food and Drug Administration, dedicated efforts have been made to utilize MSNs to construct nanoplatforms for drug delivery and cancer chemotherapy (Li T. et al., 2019). MSN DDS design has been extremely versatile. Some researchers have used active targeting groups to improve MSNs tumor targeting and improve chemotherapy selectivity (Cheng et al., 2015; Chen L. et al., 2016; Murugan et al., 2017), while others rely on the characteristics of the tumor microenvironment, such as lower pH and higher glutathione (GSH) content than normal cells. Many pH and/or redox responsive MSNs to release chemotherapeutic drugs have been designed (Cheng et al., 2017; Murugan et al., 2017; Cheng Y.-J. et al., 2019). Enzyme, thermal, and ultrasound responsive MSN DDS have also been studied (Chang et al., 2013; Li X. et al., 2018; Zhu et al., 2019). More recently, dual therapeutic agents co-delivered by MSNs to exert synergistic action and improve the effect of chemotherapy have been investigated (Zhang Y. et al., 2015; Murugan et al., 2016; Wang L. et al., 2018; Li X. et al., 2019; Xing et al., 2020) (Figure 1).

The mechanisms of cancer occurrence involves multiple pathways (Xu et al., 2015; Qiu L. et al., 2018), therefore it is unlikely that a single therapeutic mechanism will be sufficient to completely eradicate cancer. In support of this supposition, every therapy mode including chemotherapy, has demonstrated drawbacks (Fan et al., 2017). Combining chemotherapy with other treatment modalities is a good strategy to combat these shortcomings and augment therapeutic efficacy. Furthermore, chemotherapeutic combination therapies can reduce drug dosage to patients, lightening side effects while enhancing efficacy (Yu et al., 2018; Shrestha et al., 2019; Zhang et al., 2019). Combination chemotherapy possesses great potential for cancer treatment (Goldin, 1980).

**Figure 2:**
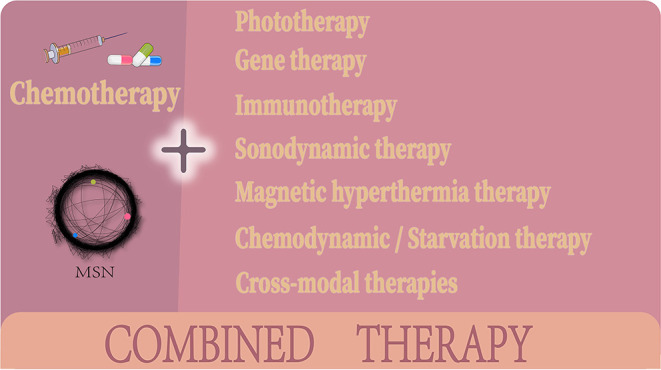
MSN nano-DDS in chemotherapeutic combination cancer therapies.

In this review, we summarize the progress made on MSN based chemo-combination therapies according to the different combination treatment modalities (Figure 2). We focus on the synergistic therapeutic effects achieved by these combined systems, emphasizing the advantages of combination therapy over monotherapy and highlighting how a successful combination compensates for the shortcomings of chemotherapy. Then, we conclude with the challenges faced by MSN based combination chemotherapy systems and what improvements are needed for these treatment systems to become mainstays in cancer therapy.

## Chemotherapy and Phototherapy

Phototherapy, including photodynamic therapy (PDT) and photothermal therapy (PTT), is a non-invasive therapeutic strategy commonly used as a supplement to chemotherapy in order to overcome the deficits of the monotherapy (Qin et al., 2018; Cheng et al., 2020). In the presence of light, photoactive therapeutics (photosensitizers) of PDT or PTT are excited to produce reactive oxygen species (ROS) or hyperthermia to kill cancer cells (Robertson et al., 2009; Chen J. et al., 2019). The high surface area, large pore size and pore volume of MSNs make them ideal candidates for multi-drug loading and therefore, a combined platform for photo-chemotherapy. In the following section we summarize photoactive mesoporous silica-based chemotherapeutic nanoplatforms according to the modality of phototherapy used in the combination (Table 1).

**Table 1:** MSN-based nanoplatforms for Chemotherapy-phototherapy and their synergistic effects.

**MSN-based nanoplatform**	**Combination therapy**	**Therapy agent**		**Chemotherapy drug**	**Synergistic effect**	**References**
BMHDC	PDT	PS	Ce6	DOX	Combination index (CI) of 0.21	Fang et al., 2019
MSN-Au-PEG			Au NPs		Cell viability of MSN-Au-PEG group ranged from 73.49 to 12.1%, lower than that of the non-illuminated group and even lower than that of free DOX group.	Guo et al., 2020
DOX@MSN-NH_2_	PTT	PS	rGO		Cell death rate: DOX@MSN@rGO-FA with NIR (PTT + chemotherapy): 68%; MSN@rGO-FA with NIR irradiation (PTT alone): 52%; DOX@MSN@rGO-FA without NIR (chemotherapy alone): 33.4%.	Wang et al., 2015
rGO@Porous Silica nanocookie				CTP	Cell death rate: Nanocookie-CPT with NIR (PTT + chemotherapy): 90%; CPT-free nanocookie with NIR (PTT alone): 60%; nanocookie-CPT without NIR (chemotherapy alone): 20%.	Chen et al., 2014
FMSN@BP-DOX-FA			BPQDs	DOX	Cell death rate: FMSN@BP-DOX-FA with NIR: 73.5%; FMSN@BP-DOX-FA without NIR: 64.78%; FMSN@BP-FA with NIR: 10%.	Qiu M. et al., 2018
GNR@SiO_2_-5-FU-ICG	PDTandPTT	PS	ICG	5-FU	Tumor growth inhibition ratio: GNR@SiO_2_-5-FU-ICG under laser (PTT + PDT + chemotherapy): 100%; GNR@SiO_2_-ICG under laser (PTT + PDT): 88.27%; GNR@SiO_2_-NH_2_ under laser (PTT alone): 69.43%; ICG-NHS under laser (PDT alone): 38.14%; 5-FU under laser (chemotherapy alone): 15.90%.	Fang et al., 2017
		PA	GNR			
MT@L-PTX@FA		PS	Te NDs	PTX	Cell viability: MT@L-PTX@FA with irradiation: ~25%; MT@L with irradiation: ~45%; MT@L-PTX@FA without irradiation: ~40%; free PTX: ~45%.	Xiao et al., 2019
		PA				

### Photodynamic-Chemotherapy

PDT is an emerging therapeutic procedure in cancer treatment that has attracted significant attention due to its high selectivity, non-invasive nature and minimal side effects, when compared to conventional therapy (Liao et al., 2016). Photosensitizer (PS) selection, tissue oxygen levels, and light wavelength are all key factors of PDT. Briefly summarized, under a specific wavelength of light, the PS transfers absorbed energy to oxygen, inducing a transformation from its triplet ground state to its singlet excited state and instigating cytotoxic effects (Leonidova et al., 2014). Due to the limited tissue penetration depths of most wavelengths used to activate PSs, PDT is non-viable for deep-seated tumors or metastasis. However, since the ROS generation from PDT has been reported to promote anti-cancer drug release (Chen Y.-W. et al., 2016; Cheng K. et al., 2019; Wong et al., 2020), a combination of PDT and chemotherapy could to enhance the therapeutic outcomes of both treatments.

MSNs have attracted substantial attention as a potential PDT partner in recent years, due to their structural merits. Many PSs aggregate easily (reducing their efficacy) and have a poor intracellular uptake, limiting their applicability in solid tumors (Ding et al., 2018; Oh et al., 2019; Fu et al., 2020). Integration with MSNs can prevent the aggregation of PSs as well as improve the targeting ability and biocompatibility of PSs, leading to reduced side-effects and stronger anticancer efficacy (Zhao et al., 2010; Yao et al., 2015; Yang G. et al., 2016). Several MSN vehicles have been reported to be able to co-deliver anti carcinogens and PSs into cancer cells (Zhang et al., 2016; Kankala et al., 2017; Chen et al., 2020; Fu et al., 2020; Wang H. et al., 2020), and these studies showed several advantages to these combination systems such as enhanced biocompatibility, improved cellular uptake of the payloads, and enhanced therapeutic efficiency (Yan et al., 2018). In one such example, Fang et al. (2019) designed a hollow MSN nanoparticles (HMSNs) based nanoplatform into which the chemotherapeutic agent DOX and photosensitizer chlorine e6 (Ce6) were co-loaded at 9.56 and 16.68% (w/w), respectively. The HMSNs-DOX-Ce6 were further modified with bovine serum albumin integrated manganese dioxide nanoparticles (BSA-MnO_2_) to construct a multifunctional therapeutic nanoplatform the authors named BMHDC. In which, BSA is intended to improve biocompatibility and tumor accumulation while MnO_2_ serves to elevate the oxygen content within the hypoxic tumors. Combination index (CI) analysis indicated a great synergy between PDT and chemotherapy in BMHDC (CI = 0.21). In another study, Guo et al. (2020) decorated MSNs with Au nanoparticles as PSs and mPEG-SH as a GSH-triggered gatekeeper to create a reduction-responsive MSN-Au-PEG nanoplatform. The spherical structure of MSN-Au-PEG was maintained, with a particle size of ~155 nm. The pore diameter of MSN-Au decreased from 3.37 to 2.67 nm after coating with mPEG-SH. The particles achieved a drug loading content (DLC) and drug loading efficiency (DLE) for DOX of 12.3 and 43.25%, respectively. Cytotoxicity assays in Hela cells demonstrated that MSN-Au-PEG@DOX with laser irradiation exhibited the lowest cell viability (30%) compared with the non-illuminated group (40%) or the free DOX group (35%). The above results indicated that the nanoplatform displayed a significant enhancement to carcinoma inhibition due to the synergistic effect of PDT-chemotherapy.

### Photothermal-Chemotherapy

PTT employs a photothermal agent (PA) to convert light energy into heat, as opposed to ROS in PDT, and induce the thermal ablation of cancer cells (Zhi et al., 2020). Similarly to PDT, PTT is a non-invasive therapeutic modality with advantages of simplicity, minimal side effects, and remote activation. However, as with PDT limited light penetration and inevitable light scattering make PTT alone insufficient to completely eliminate tumors (Li Z. et al., 2018). Since the hyperthermia produced by PTT can enhance cellular metabolism and cell membrane permeability, the concept behind combination photothermal-chemotherapy is to not only improve the uptake of chemotherapy drugs but also prevent tumor recurrence (Zheng et al., 2013; Wang X. et al., 2017). As tumors have a higher sensitivity to many chemotherapeutics at elevated temperatures (Hauck et al., 2008), the cytotoxicity of chemotherapy can be increased, thereby the dosage of anticancer drugs can be reduced and systemic side-effects minimized (Yang Y. et al., 2017).

In recent years, MSNs have emerged as powerful candidates for DDSs and they have been widely used for the codelivery of PA and chemotherapy drugs as a combination therapy (Shu et al., 2018; Tian et al., 2018). Copper sulfide nanoparticles (CuS NPs) (Chen F. et al., 2015; Zhang et al., 2015a,b; Zhang Y. et al., 2015; Peng et al., 2017; Wang F. et al., 2018; Li et al., 2020), polydopamine (PDA) (Zhang et al., 2018; Chen C. et al., 2019), gold nanorods (AuNR) (Zhang et al., 2012; Liu et al., 2015; Huang et al., 2017; Ramasamy et al., 2018; Sun X. et al., 2018; Wang Y. et al., 2019), gold shells (Rahman et al., 2017), reduced graphene oxide (rGO) (Liu et al., 2019), GO (Tang et al., 2015; Tran et al., 2018) and carbon dots (CDs) (Singh et al., 2016; Zhang et al., 2020b) are common PAs introduced in the PTT-chemotherapy systems. Wang and coworkers (Wang et al., 2015) developed a DOX-loaded amino-modified MSNs (DOX@MSN-NH_2_) with the DOX loading content of 20.9 wt% and modified with reduced graphene oxide (rGO) as a heating gatekeeper coat to achieve a multifunctional DDS. rGO possess a strong NIR absorption at 980 nm (a wavelength with good tissue penetration) and acts as the PA in this work, converting NIR light energy intothermal energy to kill cancer cells. This nanocomposite was able to kill 68% of HEp-2 cells in synergistic therapy, compared with 54% in PTT and 33% in chemotherapy alone. This *in vitro* result illustrates that the combination of PTT and chemotherapy enables a better therapeutic outcome than the monotherapies. Another rGO and MSNs based nanoplatform (162 nm) loaded with (S)-(+)-camptothecin (CPT) for PTT-chemotherapy was also reported to have a great synergistic effect. While DOX killed 33.4% of cells, and PTT killed 52% of cells, their combination was able to kill 68% of cells (Chen et al., 2014).

Black phosphorus (BP) is a new PTT agent featuring low cytotoxicity, good biocompatibility, and efficient photothermal performance (Qiu M. et al., 2018). In one study utilizing this new PA, Ren et al. (2020) constructed a MSNs based platform (150 nm) loaded with DOX and black phosphorus quantum dots (BPQDs) together. The *in vitro* results showed that the multimodal therapy of PTT and chemotherapy could induce a higher cell death rate (73.5%) in tumors compared to chemotherapy alone (64.78%).

### Photodynamic-Photothermal-Chemotherapy

**Figure 3:**
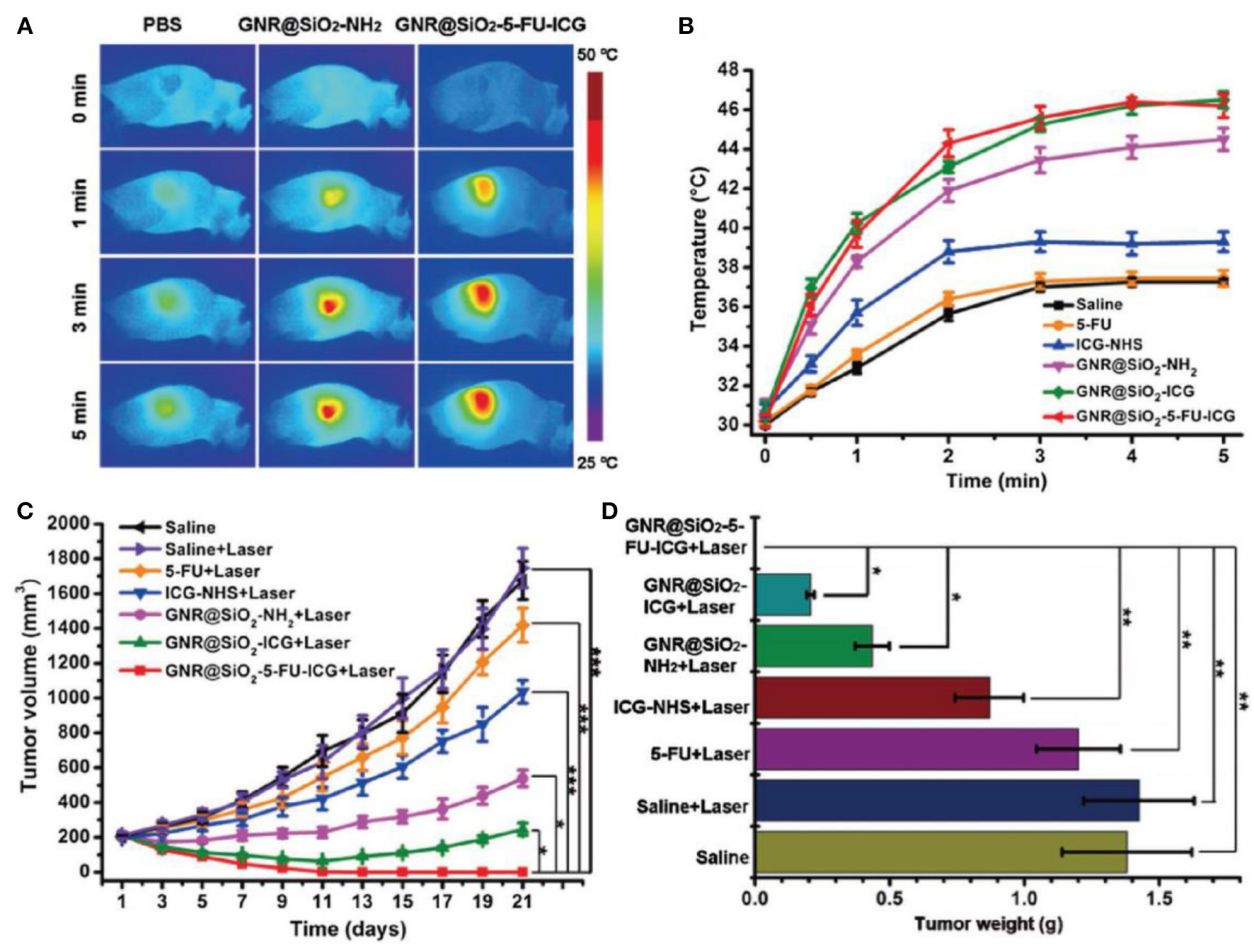
**(A)** Infrared thermal images of A375 tumor-bearing mice with different treatments at different time points upon 808 nm laser irradiation 24 h post-injection. **(B)** Temperature changes in the tumor region of the A375 tumor-bearing mice treated with saline, 5-FU, free ICG-NHS, GNR@SiO_2_-NH_2_, GNR@SiO_2_-ICG, and GNR@SiO_2_-5-FU-ICG, which irradiated at 24 h post-injection (808 nm, 1.0 W cm^−2^, 5 min). **(C)** Tumor growth of mice received different treatments. **(D)** Tumor weights of nude mice on day 21 after different treatments (Fang et al., 2017). Copyright 2017, Wiley. *p < 0.05; **p < 0.01; ***p < 0.001.

Since both PDT and PTT are triggered by light irradiation, integrating both methods with chemotherapy into a trimodal nanosystem seems a viable approach. Indeed, this combination has already proven to have superior therapeutic efficacy than any mono or dual therapy (Yang D. et al., 2016) and there have been numerous attempts to integrate both of the phototherapeutics and chemotherapeutics into MSN-based single formulation (Luo et al., 2016; Sun Q. et al., 2018; Yan et al., 2020). Fang et al. (2017) synthesized mesoporous silica-coated gold nanorods (100 nm) loaded with 5-fluorouracil (5-FU) and conjugated to indocyanine green (ICG). With 5-FU, ICG, and the gold nanorods (GNR) responsible for the chemotherapy, the PDT and the PTT, respectively. The addition on an MSN coating was able to improve both the photostability and the loading capacity of the GNR. The as-synthesized GNR@SiO_2_-5-FU-ICG realized a trimodal synergistic therapy of PDT, PTT and chemotherapy under multimodal imaging guidance. Quantitative tumor growth inhibition ratio in nude mice treated by GNR@SiO_2_-5-FU-ICG under laser irradiation was 100%, while those treated with GNR@SiO_2_-5-FU under laser and GNR@SiO_2_-NH_2_ were 88.27 and 69.43%, respectively (saline groups were regarded as 0%) (Figure 3). The nanoplatform was able to completely eradicate tumor without recurrence, demonstrating the superiority of the combination therapy. Wen and coworkers (Xiao et al., 2019) also designed a trimodal nanoplatform (250 nm) by introducing tellurium nanodots (Te NDs) into MSNs through *in situ* formation and then loading the system with paclitaxel (PTX). Here, the Te NDs work as both PS and PA concurrently, producing ROS and heat under NIR irradiation. When the concentration of PTX was 80 μM, MTT assay showed that HepG2 cells treated with MT@L-PTX@FA under irradiation had the lowest cell viability (~25%), significantly out performing MT@L with irradiation (~45%), MT@L-PTX@FA without irradiation (~40%), and free PTX (~45%). The results prove that this synergistic approach was able to enhance therapeutic outcomes.

## Chemotherapy and Gene Therapy

Traditional cancer therapies focus on killing cancer cells directly, which can achieve short-term effects but has little effect on drug resistance or metastasis and so does little to prevent tumor relapse. The occurrence of cancer is closely related to gene structure and function changes, which provides us with another strategy, gene therapy. With the sequencing of human genome, gene therapy has made noteworthy progress in the past few decades. In particular, the combination of gene therapy and chemotherapy has been widely studied and has been proven to enhance therapeutic efficiency and reduce side effects, achieving a synergistic effect in cancer treatment (Shen et al., 2014). While promising, a crucial step for this combination therapy is the development of suitable carriers for the precise delivery and controlled release of gene therapy agents such as plasmids, DNA, small interfering RNA (siRNA), micro RNA (miRNA), and short-hairpin RNA (shRNA). MSNs are easily functionalized with positively charged polymers to enable electrostatic interactions with nucleic acid and their cavities are able to load chemotherapeutic drugs effectively. As such, they are an extremely promising carrier for gene/drug codelivery (Table 2).

**Table 2:** MSN-based nanoplatforms for Chemotherapy-gene therapy and their synergistic effects.

**MSN-based nanoplatform**	**Gene**	**Chemotherapy drug**	**Synergistic effect**	**References**
MSN-SS-CP	p53	DOX	Treated with p53, DOX, p53/DOX, HELA cells apoptosis rate: 15.5, 22.6, 42.1%	Lin et al., 2017
DS-DOX-PEGA			Treated with p53, DOX, p53/DOX, relative tumor volume: 6.0, 4.5, 1.9	Zhang et al., 2017
MSN-g-PCAAMC-b-PDMAEMA		5-FU	Treated with p53, 5-FU, 5-FU/p53, MCF-7 cells apoptosis rate: 9.41, 14.32, 21.33%	Zhou et al., 2020
PMSNs	HNF4α-encoding plasmid	Cisplatin	Treated with HNF4α/cis, the growth of Huh7 cells was about 6, 3-folds decreased than HNF4α, cis singly	Tsai et al., 2019
DPSN	Bcl-2 siRNA	DOX	Treated by siRNA, DOX, siRNA/DOX, HELA cells viability: 33.5, 39.4, 16.6%	Lee et al., 2018
MSN-COOH@ZIF-8			Treated by DOX, siRNA/DOX, MCF-7/ADR cells apoptosis: 36.3, 88.2%	Pan et al., 2018
MSNs-SS-siRNA@DOX			Treated with DOX, siRNA/DOX, tumor growth inhibition: 85.2, 96.4%	Zhao et al., 2017
MSNs-PPPFA			Treated with DOX, siRNA/DOX, MDA-MB-231 cells apoptotic rate: 22.51, 36.88%	Zhou et al., 2016
MSNs	Survivin siRNA	ETO/DOC+CAR	Treated with DOC+CAR, IC_50_ in A549 cell: 1.66, 0.85	Dilnawaz and Sahoo, 2018
MSN-FA	MRP-1 siRNA	Myricetin	Treated with Myr/siRNA, tumor weight was about 1/4 of treated with Myr	Song et al., 2020
CP-MSNP@DOX/siRNA	PKM2 siRNA	DOX	Compared to monotherapy, combination therapy resulted in an almost 3-fold decrease in the tumor weight	Shen et al., 2017
MSNs@MONs	p-gp siRNA	DOX	Treated with DOX, H-MSNs-DOX, H-MSNs-DOX/siRNA, inhibition rate of tumor growth: 50.7, 76.8, 87%	Sun et al., 2017
MSNCs	T-type Ca^2+^channel siRNA	DOX	Treated with pMSNC/siRNA, pMSNC/DOX, pMSNC/DOX/siRNA, inhibition rate of tumor growth: 47, 45.5, 76%	Wang S. et al., 2019
MSN-SS-PEI	shABCG2	DOX	Treated with DOX, DOX/shRNA, CSC ratio: 1/2368, 1/57193	Chen Z. et al., 2016
MCP	P-gp shRNA	DOC	Treated with DOC/shNC, DOC/shRNA, HepG2/ADR cells apoptotic rate: 28.05, 62.93%	Wu et al., 2018
Dm@TMSN	miRNA-145	DOX	Treated with DOX, miRNA, DOX/miRNA, tumor weight: about 140, 100, 30 mg	Liu et al., 2018
MSNPs	miR211	TMZ	Treated with TMZ, miRNA, TMZ/miRNA, T98G cells apoptotic rate: 49.1, 36.88, 70.86%	Bertucci et al., 2015

p53 is a tumor suppressor gene, the disfunction of which has been found to have the highest correlation with human tumors, making it an ideal target for combination chemo-gene therapy. Lin et al. (2017) conjugated chitosan with poly (amidoamine) (PAMAM), which can absorb the p53 plasmid and then modified the chitosan derivatives onto the surface of MSNs to be the gatekeeper of DOX loaded into the pores (average diameter 2.3 nm) by a redox-responsive disulfide bond. The size of the nanosystem was about 100 nm, which is suitable for cell uptake. The nanocarrier proved to have excellent DOX/p53 codelivery ability and showed a satisfactory transfection efficiency (27.6%), very close to PEI −25 k (29.8%) *in vitro*. Importantly, the drug/gene dual delivery nanosystem showed a better inhibition for Hela cell (36% cell viability) than the drug (51%) or gene (75%) used alone, exhibiting the synergistic effect of chemotherapy and gene therapy. Zhang et al. (2017) also constructed a redox-responsive silica-based nanosystem which enable codelivery of DOX and p53. The primary difference being that the disulfide bond was directly inserted into the silica backbone and covalently linked to DOX by the one pot method, allowing the nanosystem to achieve redox-responsiveness, controlled release, and self-degradation. Recently, a smart drug/gene nanocarrier was developed by Zhou et al. (2020), which utilized UV crosslinked/pH de-crosslinked coumarin as the gatekeeper of MSNs loaded with the chemotherapy drug 5-FU and p53 carried by cationic poly(glycidylmethacrylate)-b-poly(2-(dimethylamino)ethylmethacrylate) (PGMA-b-PDMAEMA). In addition to achieving a synergistic effect of chemotherapy and gene therapy (21.33% apoptosis rate of cancer cells, compared to 14.32% for 5-Fu and 9.41% for p53 monotherapies), coumarins can also emit blue fluorescence, enabling the nanocarrier to function as a fluorescent probe to detect trace drugs concentrations.

In addition to p53 which is associated with most cancers, there are also genes associated with specific cancers. Hepatocyte nuclear factor 4α (HNF4α) is an important transcription protein that regulates the differentiation of hepatocytes and maintains the biological function of hepatocytes. Based on this, Tsai et al. (2019) investigated an approach to deliver the gene encoding HNF4α and the chemotherapeutic drug cisplatin to hepatocellular carcinoma (HCC), via polyethyleneimine-modified MSNs (PMSNs). After treatment with PMSN/HNF4α plasmid DNA/cisplatin, HNF4α in Huh7 cells was over expressed and resulted in the proportion of CD133 enriched cells decreasing significantly.

Since Fire et al. (1998) first proposed RNA interference (RNAi) technology its application in cancer therapy has grown rapidly. RNAi molecules include siRNA, shRNA and miRNA, of which siRNA have been studied most. siRNA is a type of chemically synthesized double-stranded RNA. It is transported into cells and then incorporated into the RNA-induced silencing complex (RISC), a protein-RNA complex which separates the strands of the RNA and discards the sense strand. The anti-sense strand then guides RISC to cut the target messenger RNA (mRNA), resulting in hindrance of the production of its encoded protein (Deng et al., 2014). Researchers have used mesoporous silica-based multifunctional carriers to deliver DOX and Bcl-2 siRNA to treat a variety of cancers (Zhou et al., 2016; Zhao et al., 2017; Lee et al., 2018; Pan et al., 2018). For example, Pan et al. (2018) developed a smart nanoplatform based on DOX loaded mesoporous silica as core and Bcl-2 siRNA loaded zeolitic imidazole framework-8 (ZIF-8) as its shell, in which ZIF-8 acted as the gatekeeper of DOX with its pH sensitivity controlling the release of DOX and siRNA. Flow cytometry analysis demonstrated that the apoptosis rate of MDR cells reached 88.2% after incubation with Dox-MSN-COOH@ZIF-8/Bcl-2 siRNA but was only 36.3% without Bcl-2 siRNA.

Survivin, which is highly expressed in many types of human tumors, is a member of the inhibitor of apoptosis protein (IAP) family. Dilnawaz and Sahoo (2018) demonstrated that the combination of the chemotherapeutic drug (etoposide or docetaxel) or the proteasome inhibitor carfilzomib with survivin siRNA could induce a 12.4 or 14.6% increase in apoptosis, respectively, in A549 cells. Considering that the overexpression of multidrug resistance protein 1 (MRP1) is significantly related to the clinical drug resistance of many kinds of tumors, Song et al. (2020) loaded MRP-1 siRNA and myricetin into MSNs and modified the nanoparticles with folic acid to target lung cancer cells. *In vitro* experiments showed that Myr-MRP-1/MSN-FA can significantly inhibit the proliferation of cancer cells and *in vivo* experiments further verified this therapeutic effect, in which the tumor volume of mice treated with Myr-MRP-1/MSN-FA decreased the most.

As precursors of siRNA, shRNA are often co-transported by MSNs-based carriers along with chemotherapeutic drugs to enhance their therapeutic effects against cancer (Li et al., 2016). Most notably, this approach is taken when reversing MDR (Chen Z. et al., 2016; Wu et al., 2018). An interesting nanovehicle was developed by Wu et al. (2018), in which they loaded the DOX prodrug with nitrobenzyl into the pores of MSNs, then covalently linked MSNs to cationic poly[2-(N,N-dimethylaminoethyl)-methacrylate] (PDMAEMA) modified by light sensitive coumarin. P-gp shRNA was then electrostatically adsorbed unto the particle surface and could be released upon activation by 405 nm light. After which, the release of DOX could be triggered by exposure to 365 nm light. In this study, the sequential release of gene agent and drug can be activated in a controllable manner via external illumination and this sequential release greatly increased the accumulation of drugs in the tumor sites, reversed MDR and improved overall therapeutic effect.

**Figure 4:**
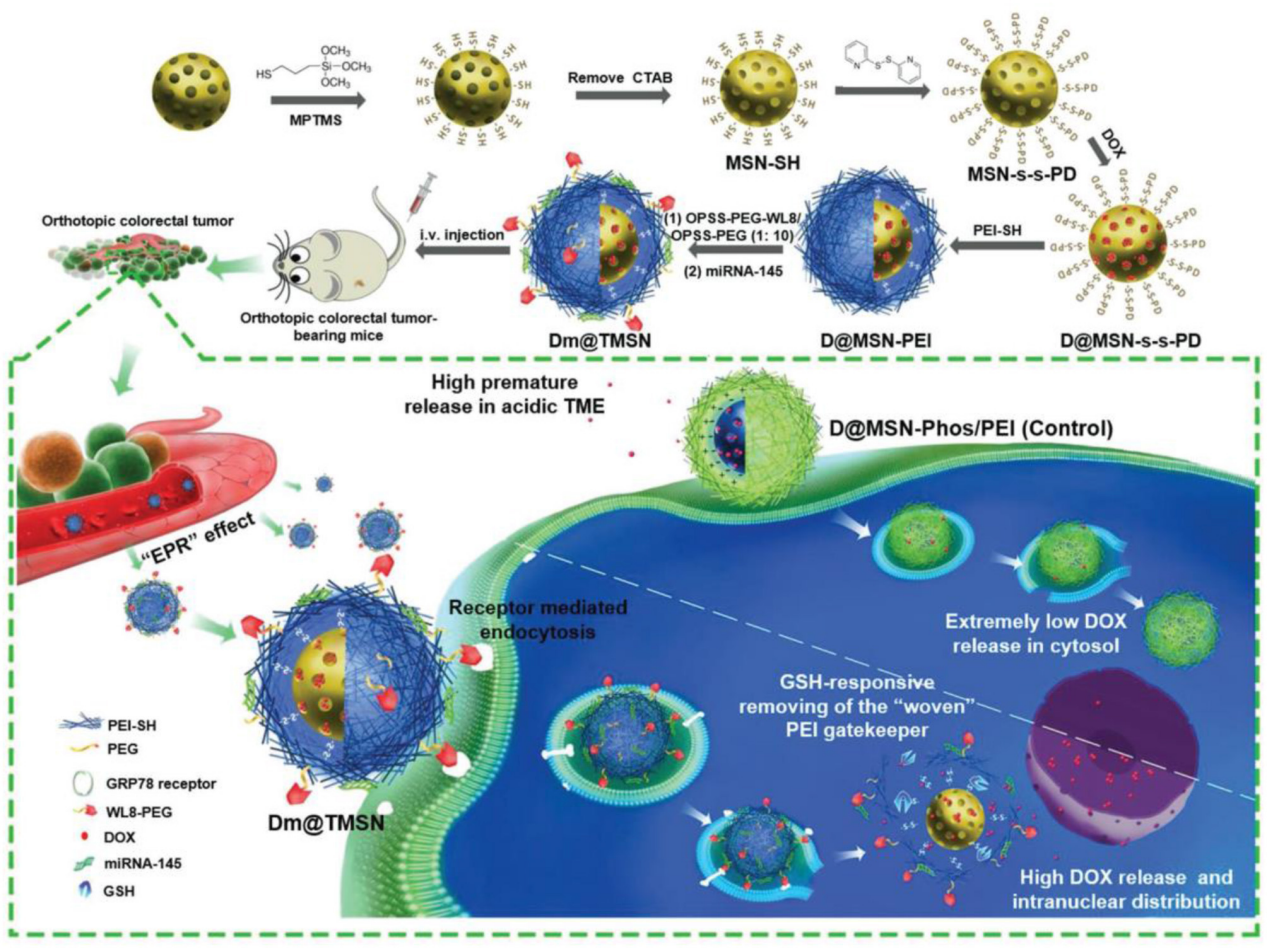
Schematic illustration of the preparation (top) and proposed mechanism (bottom) of the MSN-based DOX and miRNA-145 smart delivery system (Liu et al., 2018). Copyright 2018, Wiley.

miRNA is a type of endogenous short RNA molecule, which can be used to regulate the cleavage of target mRNA post-transcriptionally or inhibit its translation (Bartel, 2004). miRNA approaches and anti-miRNA approaches have been applied in cancer therapies, the effectiveness of these methods mostly associated with the efficacy of gene vectors. Liu et al. (2018) developed a smart silica-based nanosystem with high efficiency loading, stimulation responsivity, active targeting, and biocompatibility. Their MSNs system (Figure 4) was composed of PEI covalently linked inside the silica cavity via disulfide bond, then electrostatically bound to miRNA-145. Meanwhile acting as the gatekeeper of DOX a WL8-PEG shell is coated on the outside of the MSNs, improving the stability and targeting to SW480 cells of the combination therapy. The nanosystem showed a remarkable antitumor effect both in *in vivo* and *in vitro* experiments, with an especially excellent antimetastatic effect in an orthotopic colorectal tumor model. The expression of miR211 is upregulated in many kinds of tumors, especially in gliomas; conversely, the downregulation of miR211 can make glioma cells sensitive to temozolomide (TMZ). Working off this, Bertucci et al. (2015) used MSNs incorporated with Cy5 to transport TMZ and anti-miR221/polyarginine-peptide nucleic acid (R8-PNA221) complex to drug-resistant glioma cells. In accelerated survival experiment, MSNs with TMZ and anti-miR211 synergistically decreased the C6 glioma cells survival rate more than the sum of the MSNs-TMZ and MSNs-PNA221. The same trend was observed in their apoptosis experiment. Several studies have indicated that anti-miRNA therapy combined with chemotherapy is a potential strategy for reversing MDR. In order to improve the sensitivity of glioma cells to TMZ, Nie et al. (2020) used 93.5 ± 6.7 nm Mn-doped MSNs to deliver TMZ and10–23 DNAzyme. In acidic and reductive environments the Mn-MSNs decompose, enabling Mn^2+^ to assist 10–23 DNAzyme in silencing the O6-methylguanine-DNA methyltransferase (MGMT) gene. Western blot experiments demonstrated the gene silencing effect of Mn-MSNs/TMZ/10-23 DNAzyme and a significant decrease in IC_50_ (>3.8-fold) validated that the MDR T98G cells became more sensitive to TMZ after chemo-gene combination therapy.

## Chemotherapy and Immunotherapy

Immunotherapy, utilizing the body's natural immune system to inhibit tumor, is also a potential treatment option for combination with chemotherapy. The immune system has the dual role of inhibiting and promoting tumor growth (Schreiber et al., 2011) and immune checkpoint therapy has become a research hotspot of cancer therapy in recent days (Dyck and Mills, 2017). Compared to other treatment modes, immunotherapy can more specifically target the primary tumor and secondary tumor metastasis, it can also prolong anti-tumor response through immune memory cells to inhibit tumor recurrence (Luo et al., 2017). However, immunotherapy has a low response rate, making it ineffective for some patients (Zheng et al., 2020). Considering the limitations of chemotherapy alone as well as immunotherapy alone mentioned above, the idea of combining chemotherapy and immunotherapy came into being naturally. Though chemotherapeutic agents can induce immunogenic cells death (ICD) (Kroemer et al., 2013), most chemotherapies would induce lymphopaenia, which hampers the anticancer immune response (ACIR) (Lake and Robinson, 2005), making the combination of chemotherapy and immunotherapy difficult to realize. Zheng's group has broken the barrier between the two therapies by designing DOX@HIMSNs, a DOX-loaded and MSN-based nanoplatform (Zheng et al., 2016). The tumor volume in 4T1 tumor bearing Balb/c mice of DOX@HIMSN group was five times smaller than that of DOX group. And the fluorescent overlap between granzyme-B and caspase-3 of DOX@HIMSN group had a Mander overlap coefficient of 0.95, which was higher than the DOX group (0.88), indicating the enhanced immunological cells killing ability of DOX@HIMSN. These results showed that the highly integrated MSNs can increase tumor cell cytotoxicity as well as stimulate ACIR, indicating the potential of MSN-based nanosystems in immunotherapy combined chemotherapy. There is increasing efforts to combine the two modalities together into an MSN-based nanoplatform to achieve an enhanced therapeutic effect (Choi et al., 2019). Kong et al. (2017) developed a HMSN-mediated nanosystem called A/D/I-dHMLB to co-delivery DOX, all-trans retinoic acid (ATRA) and interleukin-2 (IL-2) for chemo-immunotherapy. A/D/I-dHMLB had a higher tumor inhibitory rate of 84.8 ± 13.0% compared to DOX group of 17.1 ± 12.4%. After the treatment of A/D/I-dHMLB, the number of myeloid-derived suppressor cells (MDSC), which impede ICD, showed a 2.7-fold decrease, while the number of mature DC and activated CD8^+^ T cell increased 14.3-fold and 3.93-fold, respectively. Other cytokines like IL-12p70 and TNF-α also increased while inhibitory cytokines like IL-10 and TGF-β decreased. All the results indicated that the design of A/D/I-dHMLB can effectively kill cancer cells and reach an enhanced antitumor immunity. Dong et al. (2017) developed a pathogen-mimicking nanocomplex (MSN-SP-LPS) with a mean hydrodynamic diameter of 167.1 ± 3.9 nm by conjugating the sodium phthalate salt of the parent LPS to MSN. The amount of TNF-α detected in RAW 264.7 cells treated with MSN-SP-LPS (~5.5 × 10^3^ pg/mL) or SP-LPS (~5.6 × 10^3^ pg/mL) was higher than that released by MSN (~0.3 × 10^3^ pg/mL) due to the presence of LPS, indicating a stronger activation of macrophages. In addition, the high amount of INF-γ secretion (900 pg/ml) provided the evidence of T cell activation, showing a strong inflammation response by MSN-SP-LPS. When treated with 1.25 μg SP-LPS/mL and 0.5 μg DOX/mL, the cell viability of splenocytes treated with MSN-DOX-SP-LPS combination was the lowest (~72%), compared to ~74% for MSN-DOX and ~95% for SP-LPS, indicating the superiority of the synergistic effect of immuno-chemotherapy.

## Chemotherapy and Sonodynamic Therapy

Sonodynamic therapy (SDT), another non-invasive therapeutic modality, has shown specific advantages in cancer therapy when compared to its counterparts like PDT or PTT, since SDT can reach deeper tumor sites due to the high tissue penetrating nature of ultrasound (US) waves (Qian et al., 2016). SDT can kill cancer cells by producing cytotoxic ROS through the combination of US with a sonosensitizer, while minimizing damage to the surrounding normal cells (Chen Y.-W. et al., 2016). Sonosensitizers include organic materials such as porphyrins (Hachimine et al., 2007; Yumita et al., 2010), erythrosine B (EB) (Yumita et al., 2002) and Rose Bengal (RB) (Sugita et al., 2015), as well as inorganic materials like titanium dioxide (TiO_2_) (Harada et al., 2011) and silicon nanoparticles (Osminkina et al., 2015). Interesting, MSNs have been shown to have impressive SDT activity due to their high porosities, which allow the free diffusion of molecules to generate ROS.

Since MSNs can simultaneously play the role of sonosensitizer and drug carrier, there is exciting potential for a synergistic nanoplatform integrating SDT and chemotherapy. While promising, SDT monotherapy still has some limitations such as lacking tumor-targeting ability, hypersensitivity to light (Lafond et al., 2019), tumor hypoxia (Zhao et al., 2020) and insufficient lethality to kill all cancer cells. The US used in SDT promotes drug release in chemotherapy (Ding et al., 2017), while the chemotherapy could compensate for the weaknesses of sonodynamic monotherapy, creating the ideal synergistic environment. Ding et al. (2017) reported a 50 nm core-shell MSN-based nanocomplex with DOX loading and targeting group methacrylated hyaluronic acid (m-HA) gel functionalization to realize a synergistic therapy combining chemotherapy and SDT. The surviving percent of cells treated by DOX@MSN-HA under US was only about 5% compared to that of DOX@MSN-HA (35%) and MSN under US (70%), highlighting the synergistic potential of SDT and chemotherapy.

## Chemotherapy and Magnetic Hyperthermia Therapy

Recently, magnetic hyperthermia therapy has been proven to be effective tool in the struggle against cancer. Magnetic hyperthermia utilizes the heat from the energy dissipation of magnetic particles to cause the irreversible necrosis of cancer cells, while leaving normal tissues undamaged (Kobayashi, 2011; Brollo et al., 2016). Additionally, magnetic hyperthermia has been shown to accelerate the release of the anticancer drug DOX from nanoplatforms, making it a potential partner to improve the efficacy of chemotherapy (Tian et al., 2018). In return, chemotherapy as a whole-body treatment can make up the limitation of magnetic hyperthermia therapy as a treatment only for local oncology. Therefore, combining chemotherapy with magnetocaloric therapy is a promising method to inhibit tumor growth and many MSN-based systems have been reported to make this a reality.

Tian et al. (2018) developed poly(N-isopropylacrylamide-co-methacrylic acid) [P(NIPAM-co-MAA)] coated magnetic mesoporous silica nanoparticles (MMSNs) with particle size 255 ± 28 nm and pore size 2.6 nm to achieve a combination chemo-magnetic therapy. Under exposure to an alternating magnetic field (AMF) at a frequency of 409 kHz and magnetic field strength of 180 Gauss, the MMSNs generated enough heat to raise the cell temperature to 64.2°C within 15 min, inducing both hyperthermia and the controlled release of loaded DOX. A CCK-8 assay showed that the cell viability of Hela cells after treatment with the synthesized DOX-MMSN@P (NIPAM-co-MAA) nanoparticles decreased to only 23%, which was significantly lower than that of cells after treatment with DOX (76%) or AMF (42%) alone. This shows the strong synergistic therapeutic effect of chemo-magnetic hyperthermia therapy and provides a promising platform for combined chemotherapy.

Iron nanomaterials are used as magnetic therapeutics in many MSN-based synergistic systems due to their strong response to AMFs (Zhu and Tao, 2015; Guisasola et al., 2018). Cai et al. (2019) successfully synthesized CSiFePNs (220 nm) by loading superparamagnetic ferroferric oxide and paclitaxel (PTX) into MSNs coated with MDA-MB-231 cell membranes. The combination system showed the highest anticancer ability (IC_50_ value of 0.8 μgL^−1^) compared to CSiFeNs with AMF (IC_50_ value of 3.6 μgL^−1^) or CSiFePNs without AMF (>0.8 μgL^−1^), further demonstrating that the combination of magnetotherapy and chemotherapy possesses great potential for the treatment of carcinomas.

## Chemotherapy, Chemodynamic Therapy, and Starvation Therapy

Chemodynamic therapy (CDT) is a novel modality to treat tumors by using transition metals to convert local hydrogen peroxide (H_2_O_2_) into highly toxic hydroxyl radicals (•OH) to kill cancer cells (Huo et al., 2017). Because CDT responds to the acidic and hydrogen peroxide rich microenvironment of tumors, it is highly selective. However, there are still challenges to face in CDT such as insufficient intratumor H_2_O_2_, inadequate H^+^, as well as the unsatisfactory catalytic capacity of chemodynamic agents (Cheng K. et al., 2020; Wang W. et al., 2020). As a local treatment modal, CDT could be a supplement to chemotherapy to enhance overall therapeutic efficacy. Combinations of chemotherapy and CDT with MSNs as the DDS have reported strong potential in anticancer treatment (Kankala et al., 2017; Zhang et al., 2020a).

Another oncotherapy strategy, starvation therapy, also responds to the tumor microenvironment, is a superb strategy to treat cancer, and may address the shortcomings of CDT (Hao et al., 2019). In contrast to normal cells, the glycolysis of cancer cells is upregulated even in an oxygen-sufficient situation (Warburg et al., 1927). Starvation therapy attempts to exploit this by utilizing glucose oxidase (GOx) to cut off nutrients to cancer cells, starving them to death. Several synergistic MSN-based nanoplatforms integrating chemo and starvation therapy have been reported (Cheng K. et al., 2019; Zhang et al., 2020b). As discussed above, CDT is limited by the concentrations of H_2_O_2_ and H^+^. Starvation therapy produces excess H_2_O_2_ and causes a decrease in pH, making it a perfect pair for CDT. Furthermore, starvation therapy itself is not enough for completely eliminate cancer cells, necessitating combination with additional treatment modalities to achieve the desired therapeutic effect (Fu et al., 2018). As both CDT and starvation therapy have been successfully integrated with chemotherapy and GOx-triggered starvation therapy can induce sequential CDT and chemotherapy, it is possible to construct an all-in-one system featuring tri-modal therapy.

**Figure 5:**
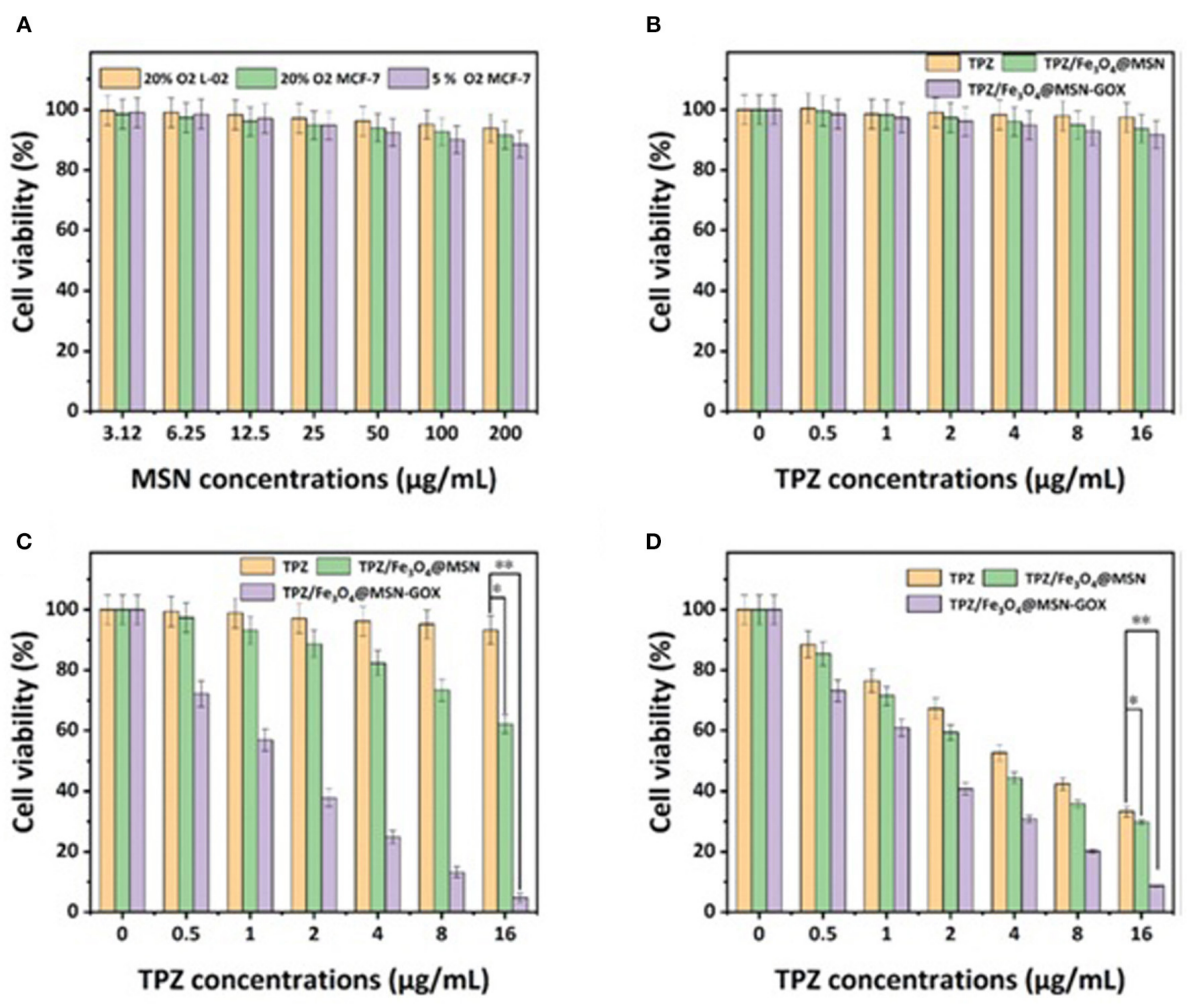
**(A)** Cell viability rates of different MSN concentrations at different oxygen concentrations. **(B)** L-02 cell viability rates of different TPZ dose-dependent concentrations of different nanoparticles in 20% O_2_ concentration with glucose (1 mg mL^−1^). **(C)** MCF-7 cell viability rates of different TPZ dose-dependent concentrations of different nanoparticles in 5% O_2_ concentration. **(D)** MCF-7 cell viability rates of different TPZ dose-dependent concentrations of different nanoparticles in 5% O_2_ concentration, which was treated by different nanoparticles calculated by the MTT assay (Cheng K. et al., 2020). Copyright 2020, RSC.

In one such example, starvation therapy and chemodynamic therapy were reported to combine with chemotherapy in a single nanosystem designed by Cheng K. et al. (2020). Hypoxic prodrug tirapazamine (TPZ) and high efficiency catalyst Fe_3_O_4_ were loaded into MSNs and the MSNs surface functionalized with GOx. The drug loading rate of TPZ and GOx achieved by the authors were 14.9 and 5.8%, respectively and the hydrodynamic diameter size of Fe_3_O_4_@MSN was 88 nm. When GOx consumes the oxygen and glucose in the tumor microenvironment, it causes increased endogenous H_2_O_2_, decreased acidity, and more extreme hypoxia. Then, through the iron ion-mediated Fenton reaction (Lafond et al., 2019) using the Fe_3_O_4_ catalyst, H_2_O_2_ is transformed into cytotoxic •OH and induces chemodynamic therapy. The hypoxia then activates the hypoxia-responsive TPZ, to kill cancer cells while avoiding healthy cells (Figure 5). Under hypoxic conditions, MTT assays showed that the cell viability of MCF-7 cells after treatment with TPZ/Fe_3_O_4_@MSN-GOX was 8.6%, while that of the TPZ/Fe_3_O_4_@MSN group was 29.9% and the TPZ group was 33.3%, indicating the as-synthesized TPZ/Fe_3_O_4_@MSN-GOX displayed excellent tumor inhibition, with the GOx-induced starvation therapy triggering synergistic enhancements with chemodynamic and chemotherapy.

## Cross-Modal Chemo-Combination Therapies

The development of multifunctional nanocarriers has enabled the development of chemotherapeutic combination therapies that include more than one other synergistic modality. In order to reverse multidrug resistance, Yang H. et al. (2017) assembled sodium alginate/chitosan polyelectrolyte multilayers onto Fe_3_O_4_/Au/MSNs loaded with DOX and photosensitizer Ce6 to adsorb P-gp shRNA. After incubation with the nanoparticles and laser irradiation, the survival rate of drug-resistant cells MCF-7/ADR was inhibited by >60% compared with any monotherapy. Demonstrating that chemo-gene-photodynamic therapy had a synergistic anti-tumor effect and the ability to reverse MDR. Additionally, Fe_3_O_4_/Au endowed the nanoparticles with dual imaging modes of magnetic resonance and CT imaging, enabling real-time guided therapy.

Gold nanotriangles are excellent radiation/PTT therapeutic agents but possess high toxicity and poor drug loading capacity (Bhattaraia et al., 2017). To overcome these limitations, a kind of “Hedgehog like” Janus gold triangle-MSNs were developed by Wang and co-works to deliver the hypoxia-activated prodrug TPZ, with surface functionalized FA-PEG to improve targeting and biocompatibility (Wang Z. et al., 2019). *In vivo* and *in vitro* experimentation revealed that FA-GT-MSNs@TPZ nanoplatforms showed superior anti-tumor effects to monotherapies alone, demonstrating that hypoxia-activated radio-chemo-photothermal therapy is a very promising strategy for cancer treatment.

Lu et al. (2020) prepared mesoporous silica nanorods of a specific width (100 nm) and precisely controlled aspect ratio (AR: length/width). They loaded DOX and GOx with AR6 into MSNs, then coated the nanoparticles with an a polydopamine (PDA) layer to absorb Siramesine, a drug that can damage lysosomes and induce apoptosis. The multifunctional nanoplatform integrated chemotherapy, PTT, CDT and ST, and targeted cancer cells with FA, exhibit in a much higher lethality to cancer cells than any single therapy. These studies highlight the potential for MSN chemotherapy combinations to go beyond a two pronged assault on cancer and incorporate a whole host of therapeutic modalities.

## Conclusions and Outlook

As a standard therapy modality for cancer treatment, chemotherapy urgently needs a more targeted drug accumulation in tumor sites and strategies to overcome MDR in order to improve its practical application in the clinic. Recently, chemotherapy-based combination therapies have become an irresistible trend due to the superiority in therapeutic efficacy compared to monotherapies. MSNs with large pore sizes, diverse functionality, ease of modification and good biocompatibility are ideal materials to realize such synergistic nanoplatforms, since they can not only serve as drug carriers but also function as therapeutic agents in therapies complementary to chemotherapy. In this review, we have discussed many MSN-based nanosystems featuring the integration of chemotherapy with other therapy modals namely immunotherapy, gene therapy, phototherapy, magnetic hyperthermia therapy and sonodynamic therapy and emphasized the effects of dual- or multi-modal therapy. As expected, most of the reported cases demonstrate that MSN mediated combination therapy achieved at least 1 + 1 > 1 effect in cellular or animal level, providing experimental evidence for further promising applications of these MSN-based delivery systems. Naturally, there is no objectively best combination, as each combination has its advantages and disadvantages. However, the chemo-immuno combination therapy may have the most promising future for further clinical translation considering that immunotherapy using PD-1/PD-L1 antibodies, CTAL-4 antibodies and CAR-T treatment has been recently revealed as a powerful clinical strategy for treating cancer.

Though MSN-based combination chemotherapies have shown preliminary success in *in vitro* and *in vivo* testing, several challenges remain before these nanoparticles can put into clinical use. As highlighted in this review, nanocomposites serve as the multimodal therapy platforms, necessitating long-term biosafety tests as well as more detailed pharmacokinetic/pharmacodynamic analyses for each participating component in the complexes. Additionally, the optimal dosing ratio between chemotherapeutics and other therapeutic agents must be investigated further. Furthermore, the integration of combination therapies should be strategic, that is, they should achieve interlocking effects and smart drug delivery and release systems to maximize the synergistic benefits. Lastly, developing simpler syntheses of MSN-based nanocomplexes is a high priority, as is improving their cancer targeting capabilities.

The existing researches about MSN-based chemotherapy combination therapies are immature, however, as our understanding of materials and diseases deepens, potential applications of MSN-based DDS broaden. It is sure that chemotherapy-based nanosystems utilizing biocompatible MSNs have a bright adaptable future and great potential for clinical translation.

## Author Contributions

YG and DG: wrote—original draft preparation. JS and QW: wrote—review and editing and funding acquisition. All authors have read and agreed to the published version of the manuscript.

## Conflict of Interest

The authors declare that the research was conducted in the absence of any commercial or financial relationships that could be construed as a potential conflict of interest.
